# Spine velocity provides more accurate assessment of curve progression than height velocity in progressive female idiopathic scoliosis undergoing bracing treatment

**DOI:** 10.1186/1748-7161-10-S1-O18

**Published:** 2015-01-19

**Authors:** Benlong Shi, Saihu Mao, Yip Benjamin, Lam Tsz-ping, Zezhang Zhu, Zhen Liu, Bangping Qian, Jack CY Cheng, Yong Qiu

**Affiliations:** 1 Spine Surgery, Affiliated Drum Tower Hospital of Nanjing University Medical School, Nanjing 210008, China; 2 Department of Orthopaedics and Traumatology, Chinese University of Hong Kong, Hong Kong, China; 3 Joint Scoliosis Research Center of the Chinese University of Hong Kong & Nanjing University, Nanjing, China; 4 Department of Public Health, Chinese University of Hong Kong, Hong Kong, China

## Objective

To calculate the spinal velocity (SV) and peak spinal velocity (PSV) in progressive idiopathic scoliosis (IS) girls and subsequently to analyze their values of utilization in the assessment of curve progression.

## Summary of background data

The height velocity (HV) is traditionally utilized to provide information of the longitudinal linear growth potential and subsequently to guide the design of treatment strategy. The existence of distal-to-proximal growth gradient in adolescents, however, has made scoliosis surgeons wonder whether SV provides superior information to HV in predicting the likelihood of significant curve progression in progressive IS patients.

## Methods

Pre-pubertal IS girls receiving standardized brace treatment and being followed up regularly were retrospectively reviewed, while only those with main curve progression of 10° or more during brace treatment were finally enrolled in this analysis. During the follow-up, the following data were collected and recorded: chronologic age, standing height, Cobb angle of the main curve, spinal length and Risser sign. The HV, angle velocity (AV) and SV of each visit were calculated. Peak height velocity (PHV) and PSV of the whole follow-up period were identified subsequently by the construction of growth velocity curves. Multiple linear regression analysis was used to analyze the contributions of each maturity assessments to AV, while logistic regression model was constructed to identify the high risk factors of AV more than 5° per year.

## Results

Thirty IS girls were included in this study. Correlation was found between SV and HV (r=0.314, P=0.001). AV was significantly correlated with SV (r=0.414, P<0.001) and HV (r=0.275, P=0.005), respectively. The multiple linear regression analysis showed that AV was influenced by SV (B=0.199, P=0.001) instead of HV (B=0.187, P=0.354). The logistic regression analysis demonstrated that PSV (OR=5.052, P=0.001) rather than PHV (OR=1.979, P=0.144) was the high risk indicator for the occurrence of AV more than 5° per year.

## Conclusions

Variations of curve progressive velocity were influenced more directly by SV rather than HV, and congruously onset of PSV were endowed with the high risk of the occurrence of AV more than 5° per year in IS girls with curve progression of 10° or more, indicating the high clinical value of measurement of spinal growth in the treatment of IS.

